# Ribosomal Initiation Complex Assembly within the Wild-Strain of Coxsackievirus B3 and Live-Attenuated *Sabin3-like* IRESes during the Initiation of Translation

**DOI:** 10.3390/ijms14034400

**Published:** 2013-02-25

**Authors:** Amira Souii, Manel Ben M’hadheb-Gharbi, Bruno Sargueil, Audrey Brossard, Nathalie Chamond, Mahjoub Aouni, Jawhar Gharbi

**Affiliations:** 1 Laboratoire des Maladies Transmissibles et Substances Biologiquement Actives (LR99-ES27), Faculté de Pharmacie de Monastir, Avenue Avicenne, Monastir 5000, Tunisia; E-Mails: benmhadhebmanel@yahoo.fr (M.B.M.-G.); mahjoub.ouni@fphm.rnu.tn (M.A.); jawhargharbi@yahoo.fr (J.G.); 2 Institut Supérieur de Biotechnologie de Monastir, Université de Monastir, Avenue Tahar Hadded, BP 74, Monastir 5000, Tunisia; 3 Laboratoire de Cristallographie et RMN Biologiques (UMR 8015), Faculté de Pharmacie, Université Paris Descartes, 4 Avenue de l’Observatoire, Paris 75270 Cedex 06, France; E-Mails: bruno.sargueil@parisdescartes.fr (B.S.); audrey.brossard@parisdescartes.fr (A.B.); nathalie.chamond@parisdescartes.fr (N.C.)

**Keywords:** coxsackievirus B3, 5′UTR, IRES, translation initiation, ribosomal complex assembly

## Abstract

Coxsackievirus B3 (CVB3) is an enterovirus of the family of *Picornaviridae*. The Group B coxsackieviruses include six serotypes (B1 to B6) that cause a variety of human diseases, including myocarditis, meningitis, and diabetes. Among the group B, the B3 strain is mostly studied for its cardiovirulence and its ability to cause acute and persistent infections. Translation initiation of CVB3 RNA has been shown to be mediated by a highly ordered structure of the 5′-untranslated region (5′UTR), which harbors an internal ribosome entry site (IRES). Translation initiation is a complex process in which initiator tRNA, 40S and 60S ribosomal subunits are assembled by eukaryotic initiation factors (eIFs) into an 80S ribosome at the initiation codon of the mRNA. We have previously addressed the question of whether the attenuating mutations of domain V of the poliovirus IRES were specific for a given genomic context or whether they could be transposed and extrapolated to a genomic related virus, *i.e.*, CVB3 wild-type strain. In this context, we have described that *Sabin3-like* mutation (U^473^→C) introduced in CVB3 genome led to a defective mutant with a serious reduction in translation efficiency. In this study, we analyzed the efficiency of formation of ribosomal initiation complexes 48S and 80S through 10%–30% and 10%–50% sucrose gradients using rabbit reticulocyte lysates (RRLs) and stage-specific translation inhibitors: 5′-Guanylyl-imidodiphosphate (GMP-PNP) and Cycloheximide (CHX), respectively. We demonstrated that the interaction of 48S and 80S ribosomal complexes within the mutant CVB3 RNA was abolished compared with the wild-type RNA by ribosome assembly analysis. Taken together, it is possible that the mutant RNA was unable to interact with some trans-acting factors critical for enhanced IRES function.

## 1. Introduction

Coxsackievirus B3 (CVB3), a member of the genus enterovirus within the family *Picornaviridae*, is an established human pathogen that causes heart disease [1–3], pancreatitis [4–7] and meningoencephalitis [8,9]. CVB3 infection of heart cells leads to cardiomyopathies [10,11]. In a mouse model, cardiovirulence of coxsackieviruses was demonstrated by extensive inflammatory lesions and necrosis of heart tissue [12–14]. These infections are difficult to treat and, to date, no approved vaccine is available. However, efforts have been made to develop a vaccine against CVB3. These include an inactivated vaccine [15], several live attenuated viruses [16–20] and DNA vaccines [21,22].

The coxsackievirus genome has a long stretch of non-coding region (5′NCR) upstream of the authentic start codon. The 741 nt 5′NCR of the CVB3 RNA is highly structured containing multiple stem-loop elements [13,23,24]. It contains an internal ribosome entry site (IRES) that directs internal initiation of translation of the coding region of the viral genome [24,25]. Ribosome binding to the IRES is mediated by an unknown number of proteins, the majority of these being cellular cytoplasmic proteins, as the infecting viral RNA is translated prior to the production of any viral proteins. It is generally believed that internal initiation by picornavirus IRESes requires most of the canonical eukaryotic translation initiation factors (eIFs) [26,27]. However, as many picornaviruses (e.g., poliovirus and coxsackievirus) induce the cleavage of the eIF4G component of the tri-molecular eIF4F cap-binding complex [28], it is clear that the requirement of IRES-mediated translation for initiation factors is distinct from that of general cap-dependent protein synthesis. In addition to the canonical initiation factors, picornavirus IRES elements need additional trans-acting factors for efficient translation initiation [25].

Since the discovery of IRES-mediated translation initiation in 1988 [29,30], the study of eukaryotic translation mechanisms has led to the discovery of processes distinct from the widely accepted view of initiation via a scanning ribosome traversing from the 5′-terminal cap structure of mRNAs to a methionine start codon 50–100 nucleotides downstream. These processes require formation of protein-RNA complexes at sites hundreds of nucleotides distal to the 5′-terminus of the mRNA. The exact determinants required for such complexes are not completely understood, but involve RNA secondary/tertiary structures, canonical components of the eukaryotic translation initiation apparatus and non-canonical RNA-binding proteins (for a review, see [31,32]). These RNA-binding proteins may facilitate ribosome recruitment directly via interaction with IRES sequences and ribosomal proteins or initiation factors. Alternatively, their mode of action may be exerted indirectly via structural changes in the mRNA that then allow binding of initiation factors and/or ribosomal subunits [31].

Translation of mRNA into protein begins after assembly of initiator tRNA (Met-tRNAi), mRNA, and separated 40S and 60S ribosomal subunits into an 80S ribosome in which Met tRNAi is positioned in the ribosomal P site at the initiation codon [33]. The complex initiation process that leads to 80S ribosome formation consists of several linked stages that are mediated by eukaryotic initiation factors [33–35]. Almost the complete set of eukaryotic initiation factors (eIFs) appears to be essential in picornavirus internal initiation [27,36–39], except the cap-binding protein eIF4E [37]. Picornaviruses lack the 7-methyl G cap structure and instead have a viral protein (VPg) that is covalently linked to the 5′-terminus of the genome [40,41]. Also, during an enterovirus infection, the virus-encoded proteinase 2A cleaves eIF4G to disrupt the cap-binding complex [42,43]. Thus, the cleavage of eIF4G acts to inhibit eIF4F-mediated ribosome scanning in cap-dependent translation.

In a previous study, the limited efficiency of the translation of *Sabin3-like* mutant of CVB3 was reported [16]. This mutant had a significantly reduced translation capacity compared with the wild-type strain. This is in accordance with previous studies concerning the S3 mutation in its natural context [44,45] or in the context of poliovirus PV1 (M) [46]. The defective translation of the *Sabin3-like* mutant was more pronounced in the CVB3 than the PV1 (M) context, despite the fact that the efficiency of translation of the wild-type CVB3 was similar to that of PV1 (M) [46]. All of these data indicate that the *Sabin3-like* CVB3 IRES mutant was more handicapped than the *Sabin 3* in PV1 (M) and the attenuated *Sabin 3* strain [16]. The poor translation efficiency of the *Sabin3-like* IRES mutant could be explicated by its inability to correctly bind some essential non-canonical translation factors during the initiation of translation. In order to test this hypothesis, formation of ribosomal complexes 48S and 80S was analyzed for both CVB3 IRESes (wild-type and *Sabin3-like*). The identity of ribosomal initiation complexes was confirmed by using stage-specific translation inhibitors.

Small molecule inhibitors of bacterial protein synthesis have served as powerful tools in the elucidation of the function of the prokaryotic ribosome. Among the known inhibitors of eukaryotic translation is cycloheximide (CHX) [47]. CHX has been shown to block the elongation phase of eukaryotic translation. It is a glutarimide antibiotic that inhibits protein synthesis by associating with the 60S ribosome and inhibiting eIF2-mediated translocation along the mRNA [48–50]. By using this antibiotic, the relative amount of RNA associated with 80S complexes is enriched [48–51]. A second inhibitor widely used for this purpose was the 5′Guanylyl-imidodiphosphate (GMP-PNP) which competes with GTP incorporated into the ternary complex. It inhibits the release of eIF2 from the small ribosomal subunit and thus prevents association of the 60S subunit. By using GMP-PNP, the formation of 80S complexes is completely abolished, while the 48S complex is slightly enriched [52].

Herein, in order to identify and compare ribosomal complexes formation within the wild-type and the *Sabin3*-*like* IRESes, we developed an RNA-based affinity purification method sensitive to translation inhibitors and suitable for isolating IRES-associated ribosomal complexes. Thus, we have analyzed 48S and 80S complex assembly through 10%–30% and 10%–50% sucrose gradients in the presence of GMP-PNP and CHX, respectively. We demonstrated that neither the 48S nor the 80S complexes were assembled in the presence of the *Sabin3-like* RNA, thereby suggesting that the association of the small ribosomal subunit within the mutant RNA was abolished.

## 2. Results

### 2.1. Amplification and Cloning of the IRES

**Figure 1:**
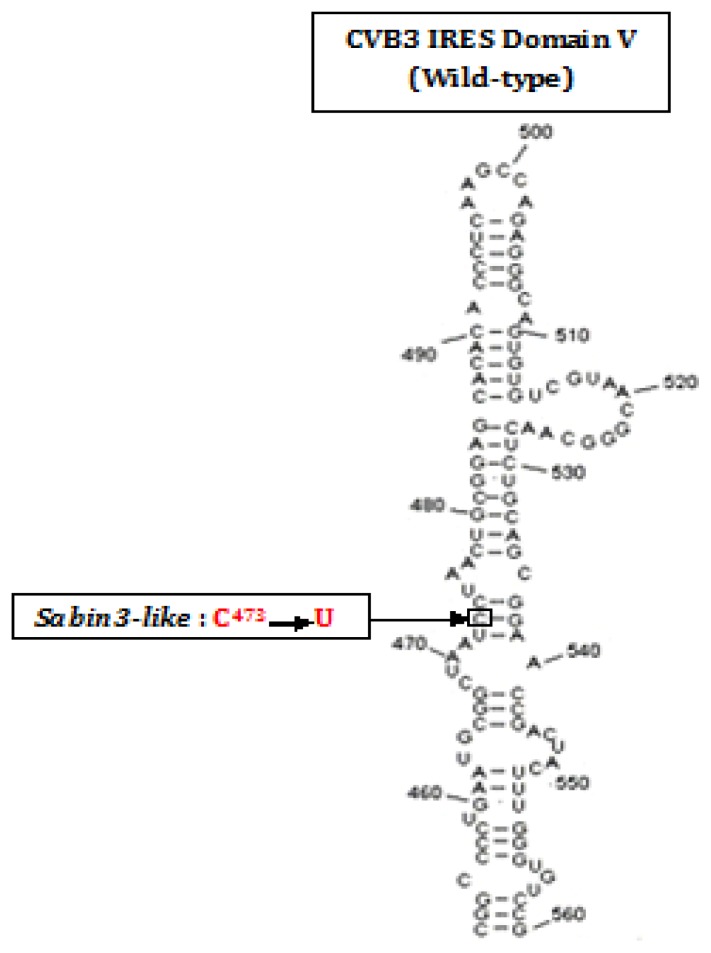
Secondary structure of domain V of the CVB3 IRES showing the region surrounding the *Sabin3-like* mutation C^473^→U.

In the present study, two CVB3 strains were analyzed: a wild-type and a mutant *Sabin3-like* strain. The attenuation of the *Sabin3-like* strain was mainly conferred by a single point mutation in the IRES domain V sequence [16] (Figure 1).

RT-PCR was performed using reverse-transcriptase MMLV and total RNA was isolated using the Trizol Reagent protocol. Both CVB3 wild-type and *Sabin3-like* IRESes were amplified using the appropriate primers. Amplified IRES DNAs (675 nt) were cloned in a pUC19 vector between EcoRI/BamHI restriction sites. Transformed pUC19/IRES clones were confirmed by colony-PCR and sequencing as described in the “Experimental Section”.

### 2.2. RNA and Fluorescent Labeling

**Figure 2:**
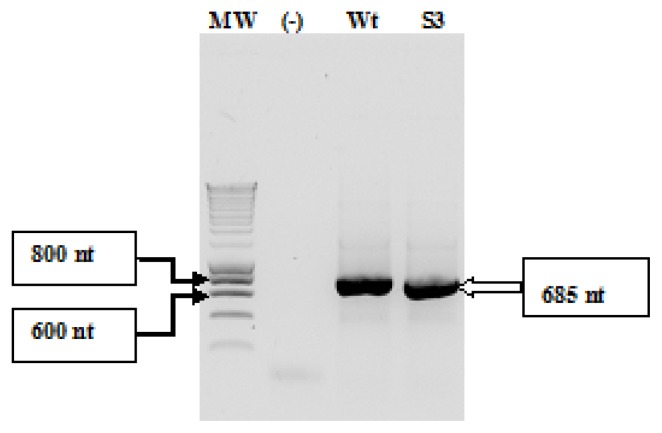
Agarose gel electrophoresis of PCR products amplified from pUC19/IRES DNAs using (T7/IRES-AUG) primers (as previously described in the “Experimental Section”). Lane MW: Smart DNA ladder 200 lanes (Eurogentec); lane (-): a negative control for the PCR reaction; lane (Wt): CVB3 wild-type IRES; lane (S3): CVB3 *Sabin3-like* IRES.

**Figure 3:**
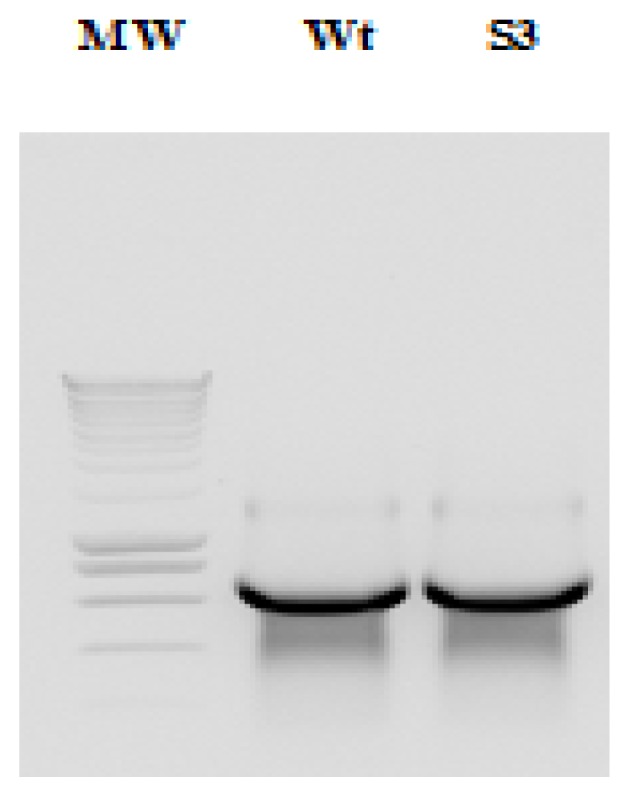
*In vitro* transcription of IRES PCR products. Lane MW: Smart DNA ladder 200 lanes (Eurogentec); lane (Wt): IRES wild-type RNA; lane (S3): IRES *Sabin3-like* RNA.

**Figure 4:**
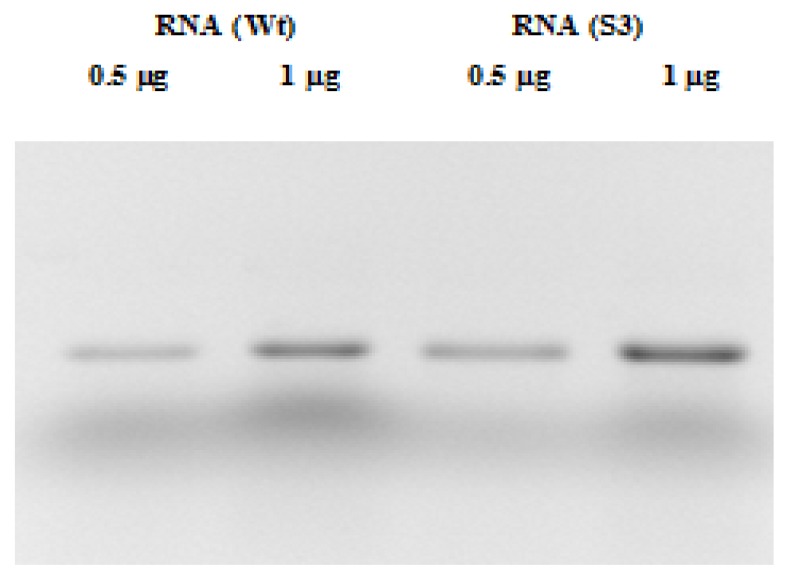
Fluorescent labeling of the IRES RNA. RNA was labeled with fluorescein-5-thiosemicarbazide and different amounts (0.5 μg and 1 μg) from fluorescent wild-type (Wt) and *Sabin3-like* (S3) RNAs were loaded on 1.5% syber-free agarose gel. This electrophoretic profile was visualized with a Luminescent Image Analyzer LAS-4000, Fujifilm using a “Fluorescence” mode.

IRES wild-type and *Sabin3-like* RNAs were transcribed using T7 RNA polymerase from PCR products amplifying the IRES containing the AUG initiator codon and 15 nucleotides from the coding region (incorporated with an appropriate primer as described above) (Figure 2). RNAs were then precipitated, purified using exclusion chromatography technique, visualized on an agarose gel (Figure 3), and then labeled with fluorescence. Labeled RNAs were loaded in a syber-free 1.5% agarose gel (Figure 4) and quantified using Biospec-NanoDrop technology.

### 2.3. Initiation Complexes Assembly

We analyzed the initiation complexes recruited on CVB3 wild-type and mutant IRESes by sedimentation on sucrose gradients. Fluorescent RNA was incubated in RRLs pretreated with a translation inhibitor, and formed initiation complexes were separated on sucrose gradients. RRLs were pretreated with either GMP-PNP or CHX. The total magnesium concentration in these binding reactions was in the physiological range and therefore allows dissociation of ribosomes to generate empty ribosomal subunits, which are, in turn, required for the association of ribosomal subunits driven by the RNA to be translated and the initiation factors involved. The ribosome assembly at the initiator AUG was blocked before or after the joining of the 60S ribosomal subunit using GMP-PNP or CHX, respectively.

**Figure 5:**
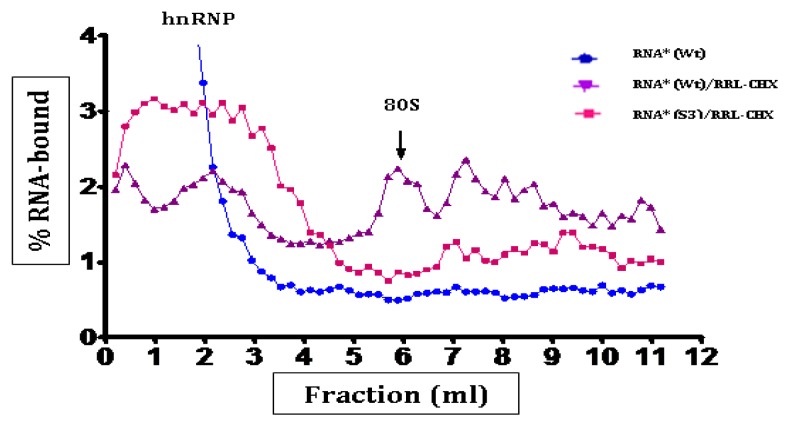
Mobility of 80S ribosomal complexes during centrifugation through 10%–50% linear sucrose density gradients. Fractionation by sucrose density gradients centrifugation of initiation complexes formed after incubation of the fluorescent RNA (RNA*) in the presence of rabbit reticulocyte lysates (RRLs) pretreated with cycloheximide (CHX). Sucrose gradients analysis of the initiation complexes was performed as described in the “Experimental Section.” Initiation complexes formed within the wild-type (Wt) and the mutant (S3) RNAs were then analyzed on 10%–50% sucrose gradients. Fractions were collected from the top, then downwards, and fluorescence was counted using a 1,420 multilabel counter, Wallac (Perkin Elmer, Life Sciences). Values obtained were extrapolated to draw a graph: % RNA-bound = f (fraction volume). The 80S peak is indicated on the graph.

**Figure 6:**
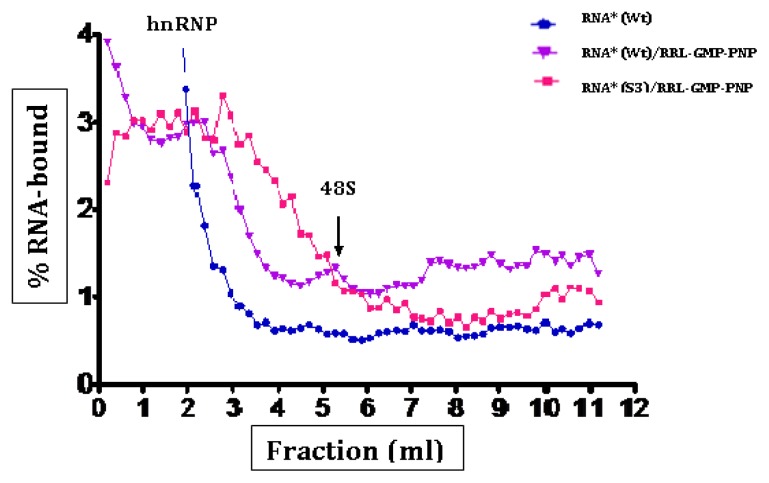
Mobility of 48S ribosomal complexes during centrifugation through 10%–30% linear sucrose density gradients. Fractionation by sucrose density gradients centrifugation of initiation complexes formed after incubation of the fluorescent RNA (RNA*) in the presence of RRLs pretreated with 5′Guanylyl-imidodiphosphate (GMP-PNP). Sucrose gradient analysis of the initiation complexes was performed as described in the “Experimental Section”. Initiation complexes formed were then analyzed on 10%–30% sucrose gradients. Fractions were collected from the top, then downwards and fluorescence was counted using a 1420 multilabel counter, Wallac (Perkin Elmer, Life Sciences). Values obtained were extrapolated to draw a graph: % RNA-bound = f (fraction volume). (Wt) and (S3) designed wild-type and *Sabin3-like* CVB3 RNAs. The 48S peak is indicated on the graph.

Ribosomal complexes (48S and 80S) were unambiguously identified by (i) comparison with the profile obtained with a control gene, (ii) modification of the profiles depending on the inhibitor used, and (iii) analysis of the profile of the same gradients which detects 40S and 60S subunits. Once formed, ribosomal complexes were separated on sucrose gradients preserving initiation complexes which had been formed with the viral RNA. The efficiency of formation of these complexes was measured by the amount of incorporated fluorescence at 254 nm. The profiles of typical gradient runs for CVB3 wild-type and *Sabin3-like* RNAs are shown in Figures 5 and 6. After separation of the initiation complexes, most of the RNA sedimented as hnRNPs (heterogeneous ribonucleoproteins), and as peaks corresponding to 48S and 80S complexes. In order to determine the exact location of those initiation complexes, the “toe-printing” approach was used.

48S complexes represent the RNA associated with the small ribosomal 40S subunit, and 80S complexes represent the RNA associated with a completely assembled ribosome also containing the large ribosomal 60S subunit. Figures 5 and 6 show that both 80S and 48S initiation complexes were formed within the wild-type RNA, but not with the *Sabin3-like* IRES. 80S initiation complexes migrate at 6 mL from the top of the gradient (Figure 5), while 48S complexes sedimented at 5.5 mL (Figure 6).

## 3. Discussion

Among the four major steps of translation (initiation, elongation, termination, and recycling of ribosomes), the rate-determining step is initiation [53,54]. Initiation of protein synthesis in the eukaryotic cell leads to the assembly of the 80S ribosome at the start codon of the mRNA. At least two mechanisms for recruiting and positioning ribosomes on the mRNA have been described [55,56]. The primary mechanism involves the recognition of the 5′ cap structure by eukaryotic translation initiation factors (eIFs) [55,56]. Alternatively, in some mRNAs (such as CVB3 RNA), a structural element named the internal ribosome entry site, allows assembly of the translational machinery [57–61]. The ribosomes are recruited upstream of the AUG triplet at 591 of the CVB3 5′UTR, also called the cryptic AUG [62–64]. IRES trans-acting factors (ITAFs) are thought to bind to the mRNA, inducing conformational changes needed to structurally form the IRES, thereby facilitating ribosome recruitment [57,59,65,66].

We have previously tried to find out if the results observed in the case of Sabin vaccine strains of poliovirus can be extrapolated to another virus belonging to the same genus of enteroviruses but with a different tropism. To test this hypothesis, we used coxsackievirus B3, known to be the most common causal agent of viral myocarditis. The introduction of the three PV *Sabin-like* mutations in the equivalent positions (nucleotides 484, 485, and 473) to the domain V of the CVB3 IRES resulted in a significant reduced viral titer of the *Sabin3-like* mutant but not on those of *Sabin1-* and *Sabin2-like* mutants [16]. Additionally, the *Sabin3-like* RNA showed a reduced efficiency of translation compared to the CVB3 wild-strain.

In the absence of an *in vitro* system using purified ribosomal subunits, initiation factors, and possibly other cellular proteins required for the formation of ribosomal initiation complexes with the CVB3 IRES RNA, we developed an assay that allowed us to determine the efficiency of ribosome association with the mutated IRES elements. In this system, we demonstrated that the efficiency of ribosome assembly with the mutated IRES was considerably impaired compared with the wild-type RNA, correlating with the most severe decrease of translation efficiency with this construct [16]. In fact, the 48S ribosomal complex of *Sabin3-like* RNA was abolished when compared with the wild-type, suggesting that the mutant prevented 48S ribosomal complex assembly on the CVB3 RNA. The 80S peak was also abolished in the presence of the mutant RNA compared with the wild-type RNA, demonstrating that neither the 48S nor the 80S complexes were assembled in the presence of *Sabin3-like* RNA. These results indicate that the serious reduction in translation efficiency observed previously with the *Sabin3-like* strain [16,17] is caused at the stage of association of the small ribosomal subunit with the viral RNA. Multiple peaks were observed, which may reflect multiple stalled 48 and 80S complexes on RNAs.

The observation that the *Sabin3-like* point mutation in domain V of the CVB3 IRES seriously affects ribosome association with the IRES, coupled with the fact that domain V is the major determinant for the binding of eIF4G and eIF4B [67], led us to the conclusion that the single nucleotide exchange may directly affect the binding of these translation initiation factors and hence impair the association of the ribosomal 40S subunit with the mutant RNA. Thus, the poor translation efficiency of the *Sabin3-like* IRES can be due to its inability to correctly bind some essential non-canonical translation factors and/or to perturbations of the secondary and tertiary structure of domain V.

Taken together, these data demonstrate that the introduction of a single substitution of the nucleotide U^473^ by a cytosine (called *Sabin3-like* mutation) affects the formation of 48S and 80S ribosomal initiation complexes. This finding correlates with previous genetic results of impaired replication of a CVB3 U^473^→C mutant [17]. Studies of recombinant poliovirus made between attenuated vaccine strains and their neurovirulent progenitors have shown that a major determinant of neuroattenuation maps to a single point mutation located within the viral IRES. Attenuated Sabin strains exhibit decreased efficiency of translation in cells of neural origin compared to wild-type neurovirulent strains [45,46]. Therefore, these strains are considered to have a neural cell-specific attenuation. It was hypothesized that the lack of yet-unidentified host cell factors could explain the tissue-specific replication of poliovirus. The molecular mechanism for the cardiotropism of CVB3 has not yet been demonstrated. In fact, the reduced viability of the *Sabin3-like* mutant of CVB3 is likely a general impairment, despite the fact that the same mutation was investigated.

The 40S ribosomal subunit has been shown to enter at a putative site 150 nucleotide upstream of the initiator AUG in the poliovirus 5′UTR [63,68,69]. The ribosome entry site encompasses a polypyrimidine tract and the conserved non-initiator AUG. The ribosome recruitment on the coxsackievirus IRES has been shown to have important determinants in the region immediately downstream of nucleotide 565 within the 5′UTR [24,70]. This region includes the pyrimidine rich tract and a nucleotide stretch 567–577 that has been shown to act as a Shine–Dalgarno-like sequence in the internal initiation of translation of CVB3 RNA [63,71]. Similar to the canonical pathway, assembly of active 80S ribosomes still requires eIF5, eIF5B, GTP and 60S subunits [39], but, in addition, also involves functional interactions of the IRES with the 40S subunit [72–74]. Bhattacharyya and Das [63] have demonstrated the putative 48S ribosomal assembly site around nt 570 on the CVB3 5′UTR, near the SD-like sequence.

Several non-canonical cellular protein factors have been shown to specifically interact with picornavirus IRES elements and influence their function, which includes La (p52), PTB (p57), UNR (p97), PCBP2 (p39), PABP [64,75–77]. Some of these factors are also involved in switching the function from translation to replication of the viral RNA. Additionally, several canonical initiation factors have been shown to interact with enterovirus IRES elements, such as eIF3, eIF4B, the cleaved part of eIF4G, *etc.* Also, several other cellular proteins have been shown to influence IRES function in general. DAP5, an 86-kDa protein, has been shown to be an ITAF for the IRES-mediated translation of its own mRNA [78].

It was reported that MFOLD structure of CVB3 IRES and its secondary structure showed a high degree of similarity to that of poliovirus 5′UTR [79] and that attenuating mutations for the Sabin vaccine strains of poliovirus are located in domain V; consequently, this domain has received a great deal of experimental attention [23]. However, the region corresponding to stem-loop (SL) G (nt 519–560) and H (nt 581–624) of CVB3 was represented with a single stem-loop (SLG, nt 559–624) in poliovirus [61,80]. Interestingly, the deletion of the first 63 nt from the CVB3 5′UTR had a negative effect on translation, whereas deletion of nt 250–529 enhanced it marginally [24,61,71]. A recent study on the secondary structure of the CVB3 5′UTR suggests long-range pairing interaction that could link domain II to domain V within the 5′UTR [23].

Involvement of the local secondary structure in modulating IRES function has been reported in some viral RNAs [63,81–83]. It has also been shown that structural organization of viral IRES depends on the integrity of a GNRA motif [81,84]. It was reported that mutation in the GNRA affects the binding at the SL-H, which might have a drastic consequence on the IRES activity [63]. Alternatively, mutations in the GNRA loop might cause partial disruption of the secondary and/or tertiary structure required for long-range RNA–RNA interactions to mediate efficient internal initiation of translation. Thus, it appears that both the sequence and the structural integrity of the apical loop at the SL-H domain might be important for interaction with a critical trans-acting factor to mediate efficient internal initiation of translation [63,85]. GNRA loop has been reported to be involved in long range RNA–RNA interaction to mediate IRES activity and has been speculated to be involved in interaction with the ITAFs [63,83].

The possible involvement of other trans-acting factors with this cis-acting element is not ruled out. Therefore, the possibility of involvement of this cis-acting RNA element with some other region of the RNA in long range RNA–RNA interactions cannot be ruled out.

## 4. Experimental Section

### 4.1. Virus

The coxsackievirus B3 (CVB3) Nancy prototype strain and the *Sabin3-like* mutant of CVB3, used as a “vaccine candidate” and obtained by direct mutagenesis [16] were used for all the experiments. These strains were propagated in Vero cells (African Green Monkey Kidney Cells) (Bio Whittaker), maintained in Eagle’s minimal essential medium (MEM), supplemented with 10% heat-inactivated fetal calf serum (FCS) (Sigma), 1% L-glutamine, 50 μg/mL de streptomycin, 50 UI/mL penicillin (Bio Whittaker), 1% non-essential amino acids (Gibco BRL) and 0.05% fungizone (Amphotericin B, Apothecon).

### 4.2. Plasmids and Cloning Conditions

The pUC19 (plasmid of University of California) (Invitrogen) was used for cloning the IRESes of both CVB3 strains. This plasmid has one ampicillin resistance gene, and an N-terminal fragment of β-galactosidase (*lacZ*) gene. The synthesis of *lacZ* fragment can be induced by IPTG (isopropyl βeta-D thio galactopyranoside). In the presence of IPTG, bacteria synthesize both fragments of the enzyme that can together hydrolyze the X-gal (5-Bromo-4-Chloro-3-Indolyl-βeta-D-Galactopyranoside) and form blue colonies. Thus, bacteria carrying recombinant plasmids cannot hydrolyze the X-gal, giving rise to white colonies.

### 4.3. Bacterial Strains

*Escherichia (E.) coli* DH5α strain was transformed with recombinant plasmids, and was cultured in Luria–Bertani (LB) broth to select transformants. This strain has many mutations that make it useful for transformation, but the most useful of these mutations is the “*lacZ Delta M15*” mutation which allows blue/white screening for recombinant cells.

### 4.4. Viral RNA Extraction

Total RNA was extracted using the Acid Guanidinium Thiocyanate Phenol-Chloroform method [86]. After ethanol precipitation, the total RNA was resuspended in DEPC (diethyl pyrocarbonate)-treated sterile water.

### 4.5. Reverse Transcription (RT)

cDNA was synthesized in 50 μL volume containing 2 μg RNA, 10 mM Tris–HCl (pH 8.3), 75 mM KCl, 2.5 mM MgCl_2_, 1 mM dNTP (Promega), 10 mM DiThioThreitol (DTT), 1 mM antisense primer IRES-R (5′- TTT gCT gTA TTC AAC TTA ACA ATg-3′), 1U RNase inhibitor (Amersham), 50 U Moloney Murine Leukemia Virus Reverse Transcriptase (MMLV-RT) (Promega). The reaction was carried out at 42 °C for 30 min followed by a denaturation step during 5 min at 99 °C.

### 4.6. Polymerase Chain Reaction

IRESes of the two studied CVB3 strains were amplified using primers IRES-F (5′- TAT gAA TTC***TAA TAC gAC TCA CTA Tag*** gTA ACT TAg AAg TAA CAC A -3′) and IRES-R′ (5′- TAT ggA TCC TTg CTg TAT TCA ACT TAA CAA TgA ATT gTA ATg TTT TAA -3′). The amplification reaction was performed in a volume of 50 μL containing 100 ng cDNA, 25 mM Tris-HCl (pH 8), 50 mM KCl, 4 mM MgCl_2_, 200 μM dNTP, 40 pmoles from each primer and 1U Taq DNA polymerase (Promega). Distilled water was included as a negative control. Amplification was initiated by a denaturation step during 2 min at 95 °C, followed by 5 cycles of 30 s denaturation at 95 °C, 30 s annealing at 48 °C, and 30 s extension at 72 °C. Then, 25 cycles of 30 s at 94 °C, 30 s at 58 °C and 30 s at 72 °C were performed. Steps were followed by a final extension (10 min at 72 °C). All PCR products were electrophoresed on 1.5% agarose gel.

### 4.7. Cloning of the IRES in the pUC19 Vector

Amplicons encoding CVB3 IRESes were double-digested with EcoRI and BamHI (Roche Applied Science) (both restriction sites were added by PCR), then purified and inserted into the pUC19 plasmid (Invitrogen) to be digested with the same enzymes. Ligation products were then transformed in chemocompetent *E. coli* DH5α cells. Transformants were selected in LB agar supplemented with ampicillin (100 μg/mL), X-gal (64 μg/mL) and IPTG (0.2 mM). Blue–white selection was used to identify the recombinant clones.

### 4.8. Screening of Recombinant Clones

To analyze the cloned sequences, randomly chosen clones were tested by colony-PCR. A single colony isolated and added into 5 μL sterile water was used as template DNA in the PCR reaction containing 15 μL premix solution. The amplification program was a pre-denaturation at 94 °C for 4 min followed by 30 cycles of denaturating at 94 °C for 1 min, annealing at 50 °C for 1 min, extension at 72 °C for 1 min, and a final extension at 72 °C for 10 min. Then, a 1.5% agarose gel was stained and DNA bands were compared to the expected sizes to determine positive clones. Finally, these clones were sequenced using an ABI Prism BigDye Terminators Sequencing Kit (Applied Biosystems).

### 4.9. RNA and *in Vitro* Transcription

The DNA sequence corresponding to the CVB3 IRES cloned in pUC19 was amplified by PCR using T7 (5′- TAA TAC gAC TCA CTA TAg- 3′) and IRES-AUG (5′- TgA TAC TTg AgC TCC **CAT** TTT gCT gTA TTC AAC TTA ACA ATg- 3′) as forward and reverse primers, respectively. Fifteen nucleotides from the coding region and the initiating codon AUG were incorporated in the reverse primer sequence. Reaction mixture components were: 50 ng linearized pUC19/IRES, 1 μM from each primer, 0.2 mM dNTPs, Taq Buffer (1X), 5U Taq polymerase and the final volume of reaction was adjusted by adding distilled sterile water. PCR was carried out following a thermal profile consisting in a pre-denaturation step during 3 min at 94 °C; 35 cycles denaturating for 1 min at 94 °C, annealing for 1 min at 50 °C, extension for 1 min at 72 °C, and a final extension step during 10 min at 72 °C.

RNA was then produced by *in vitro* transcription of PCR products containing the T7 promoter sequence. The reaction was conducted in a final volume of 100 μL using the T7 RNA polymerase (New England Biolabs), 5 mM DTT, 4 mM NTP’s, Transcription Buffer (1×) (40 mM Tris-HCl pH 8.0; 25 mM MgCl_2_; 1 mM Spermidine) and 0.04 U RNase inhibitor (Applied Biosystems/Ambion) and incubated 2 h at 37 °C. During this incubation, and when a precipitate of RNA appears, 1 U of Pyrophosphatase Alkaline (New England Biolabs) was added. Upon synthesis, RNAs were treated with RQ1 RNase-free DNAse (Promega) for 30 min at 37 °C.

RNA was then precipitated with 2.5 M Lithium chloride (LiCl), incubated for 30 min at −20 °C, centrifuged at 13,000 rpm, 30 min at +4 °C, washed with 75% ethanol, resuspended in 50 μL of nuclease-free water and purified by size exclusion chromatography. RNA concentration was then determined spectrophotometrically (NanoDrop Technology) and RNA integrity was monitored by electrophoresis on an agarose gel.

### 4.10. Periodate Oxidation and Fluorophore Coupling

Many single-molecule experimental techniques exploit fluorescence as a tool to investigate conformational dynamics and molecular interactions or track the movement of proteins in order to gain insight into their biological functions. In this context, to assess the efficacity of formation of ribosomal complexes during the initiation of translation of CVB3, the IRES RNA was labeled with fluorescein which is a synthetic organic compound available as a dark orange/red powder. Herein, the 3′terminus of IRES wild-type and *Sabin3-like* RNAs was oxidized into dialdehyde by sodium periodate and then labeled with fluorescein-5-thiosemicarbazide. The detailed procedure was as follows: RNA was oxidized using sodium periodate (0.1 M) with sodium acetate (pH 5; 0.1 M) and incubated at 25 °C during 90 min. To stop the reaction, potassium chloride (250 mM) was added. The mix was incubated on ice for 10 min, then, centrifuged at 13,000 rpm for 5 min at +4 °C. The supernatant was purified using Sephadex G50 column (GE Healthcare) to remove the excess of oxidant. Coupling was performed by addition of 5 mM fluorescein-5-thiosemicarbazide in the presence of sodium acetate (pH 5; 0.1 M), followed by incubation at 25 °C for 4–17 h in the dark.

Following a fluorescent labeling reaction, it is often necessary to remove any non-reacted fluorophore from the labeled target molecule. This is usually accomplished by size exclusion chromatography, taking advantage of the size difference between fluorophore and labeled RNA. Taking into account that fluorophores may interact with the separation matrix and reduce the efficiency of separation, specialized dye removal columns that account for the hydrophobic properties of fluorescent dyes are sometimes used. G50 Sephadex columns purification (Roche Applied Science) were used for this purpose.

Then, fluorescent-labeled RNA was precipitated by adding LiCl (7.5 M) and 2.5 vol ethanol, standing at −20 °C for 30 min, and centrifuged (13,000 rpm at 4 °C) for 20 min. The precipitate was washed with 75% ethanol and RNA was resuspended in nuclease free water. The excess of fluorophore was removed by G50 Sephadex column purification (Roche Applied Science).

### 4.11. Mobility of Initiation Complexes during Sucrose Density Gradients Centrifugation

Initiation complexes were analyzed on 10%–50% and 10%–30% sucrose gradients. Sucrose gradient analysis was used to distinguish between 48S and 80S complexes. This analysis was facilitated by inclusion of 5′-Guanylyl-imidodiphosphate (GMP-PNP) (a non-hydrolyzable GTP analog) that inhibits 60S subunit joining leading to a build up of 48S complexes and cycloheximide (CHX) that blocks the translational elongation process, leaving either the 48S or 80S complex stalled at the start codon, respectively. Rabbit reticulocyte lysates that contain cellular components necessary for protein synthesis (tRNA, ribosomes, amino acids, initiation, elongation and termination factors) were used as an *in vitro* translational system.

Ribosomal complexes were assembled on fluorescein-labeled IRES RNA. At first, 5 pmol of RNA were heated for 2 min at 80 °C, slowly cooled to room temperature in a mix containing: 25 mM magnesium acetate, 4 mM potassium acetate, 20 mM amino acid and 8 U RNAse inhibitor (Promega) for 10 min. RRLs (Promega) were preheated with either 2 mM GMP-PNP or 4 mM CHX for 10 min at 30 °C. Then, RNA was incubated with pretreated RRLs at 30 °C during 20 min. Reactions were stopped on ice, then were layered over 10%–30% (RNA/RRL+GMP-PNP) or 10%–50% (RNA/RRL+CHX) sucrose gradients (1 M Tris pH 7.6; 1M MgCl_2_, 3M KCl; sucrose 60%; 1 M DTT and H_2_O) and sedimented by ultracentrifugation at 37,000 rpm in a SW40 rotor of a Beckman Coulter ultracentrifuge for 3 h at 4 °C. Fractions were then collected using a fraction collector FC 204 (Gilson). The amount of fluorescence in each fraction was determined by a 1420 multilabel counter, Wallac (Perkin Elmer, Life Sciences) using absorption at 254 nm to detect ribosomes. The obtained values were then extrapolated to draw a graph: % RNA-bound = f (fraction volume). Reported values are the average of three repetitions with standard errors.

## 5. Conclusion

In summary, we have used in the present study a cell-free protein synthesis as the indicator of ribosome assembly initiation complexes in the presence of specific inhibitors of protein synthesis. We have demonstrated that the reduction in CVB3 translation efficiency caused by the single nucleotide exchange in the IRES of the *Sabin3-like* strain can be mediated by the impaired binding of standard translation initiation factors to domain V of the CVB3 IRES. This, in turn, causes impaired association of ribosomes within the viral RNA, and thus may contribute to the attenuated phenotype of the CVB3 mutant strain. Hence, it will be interesting to identify the specific site of the inhibition of initiation factors binding to the mutant CVB3 RNA during the initiation of translation process.
